# Diseases of the Eye

**Published:** 1882-06

**Authors:** 


### BOOK NOTICES, REVIEWS, ETC.

A Treatise on Diseases of the Eye, Etc. By Henry C. Angell, M. D., Professor of Ophthalmology in the Boston University School of Medicine, etc., etc. 6th edition, remodelled and illustrated. Bœricke & Tafel: New York and Philadelphia. 1882. pp. 404. Price, $3.00.

In the preface to this edition the author states "the main purpose of this work is to make the nature and diagnosis of ophthalmic affections comprehensible to the non-specialist, and, after this, to teach how to avoid the bad and how to avail of the good." * * * In the preface to the first edition he quotes Bæhr, who at the time that was written lamented the obstacles to the successful homœopathic treatment of diseases of the eye, "chiefly those arising from a lack of hospitals and clinical teaching," also, that "an occulist would still have a great many difficulties to contend with, which are inherent in our materia medica." This in 1870. In 1882 we turn to p. 108 of this work and find advised for the treatment of hyperæmia of the conjunctiva "a few drops of tincture of Hamamelis, or tincture of Opium, or of Hydrastis, in a cup of water, or a grain or two of Sulphate of Zinc, or of Acetate of Lead, *any of these* (we italicize), adhering, of course, to the one which seems most grateful, may be used as a fomentation, either warm or cold, to the lids several times a day." Then, on p. 13 are to be found for the treatment of catarrhal conjunctivitis such applications as Zinc. sulph., Alum, Borax, Nitrate of Silver, etc. So on throughout the compilation we find a professed homœopath resorting to allopathic heroic treatment and then we shudder for the fate of those who shall be unfortunate enough to fall into the hands of students who have sat under such teachings and who endeavor to put them into practice. This is "avoiding the bad" with a vengeance; if it is "availing of the good," our prayers are for those who resort to nothing better. What a progressive mode of correcting the "many difficulties which are inherent in our materia medica!" crab-like progress. The best portion of the book contains nothing but what is found better given in such works as Wells', Landalts', and works of that class, written by men who have something original to say and who adhere to principle. This before us is book-making pur et simple, particularly the latter, and we hope that all Hahnemannians will possess it for the purpose of seeing how some of the professed followers of Hahnemann can send out a book that purports to be homœopathic and yet contains advice that is wholly antagonistic to and at variance with the teachings of that philosopher, Take Part I of Norton's "Ophthalmic Therapeutics" and make yourself a repertory and with Berridge's Eye Repertory you will possess an armament that will be invincible in diseases of the eye and thus you *will* "avail of the good," and your patients will rise up and call you blessed. G. H. C.

